# Evaluation of liver function using liver parenchyma, spleen and portal vein signal intensities during the hepatobiliary phase in Gd-EOB-D TPA-enhanced MRI

**DOI:** 10.1186/s12880-020-00519-7

**Published:** 2020-10-20

**Authors:** Ming Yang, Yue Zhang, Wenlu Zhao, Wen Cheng, Han Wang, Shengren Guo

**Affiliations:** Department of Radiology, The Second Affiliated Hospital of Soochow University, Sanxiang Street No. 1055, Suzhou, 215004 Jiangsu China

**Keywords:** Gd-EOB-DTPA, Magnetic resonance imaging, Liver function, Portal vein, Spleen

## Abstract

**Background:**

Previous studies have used signal intensity (SI) to reflect liver function. However, few studies have evaluated liver function via the portal vein. Regarding the SI of the liver, spleen, and portal vein, no study has indicated which can best reflect liver function. Therefore, the aim of this study is to investigate whether these parameters can evaluate liver function in patients with cirrhosis and determine which is the best parameter.

**Methods:**

120 patients with normal livers (n = 41) or Child–Pugh class A (n = 50), B (n = 21) or C (n = 8) disease who had undergone Gd-EOB-DTPA-enhanced MRI were retrospectively reviewed. Comparisons of the MRI data (liver parenchyma SI, portal vein SI, and spleen SI and liver-to-portal vein contrast ratio (LPC), liver-to-spleen contrast ratio (LSC), and portal vein-to-spleen contrast ratio (PSC)) in the 15-min hepatobiliary phase images were performed among the groups, and the correlations among the liver function parameters (total bilirubin, direct bilirubin, indirect bilirubin, aspartate aminotransferase, alanine aminotransferase, albumin, creatinine, platelet count, prothrombin time and international normalized ratio), liver function scores and MRI data were also quantitatively analysed.

**Results:**

Significant differences were observed in the liver parenchyma SI, LPC and LSC among the groups. These values all decreased gradually from normal livers to Child–Pugh class C cirrhotic livers (*P* < 0.001). The portal vein SI constantly and slightly increased from normal livers to Child–Pugh class C cirrhotic livers, but no differences were found among the groups in the portal vein SI and PSC (*P* > 0.05). LPC showed a stronger correlation with the Child–Pugh score and MELD score than LSC and the liver parenchyma SI. The order of the AUCs of these parameters, from largest to smallest, was as follows: LPC, LSC, and liver parenchyma SI (*P* > 0.05).

**Conclusion:**

The liver parenchyma SI, LSC and LPC may be used as alternative imaging biomarkers to assess liver function, while the portal vein SI and PSC do not reflect liver function. Furthermore, LPC values can more effectively distinguish severity among patients with cirrhosis than the liver parenchyma SI and LSC.

## Background

The assessment of liver function is one of the most important issues in patients with cirrhosis. The Child–Pugh score and model for end-stage liver disease (MELD) score are commonly used clinically. However, these scores all have some design flaws. The five indicators (total bilirubin (TB), albumin, creatinine, prothrombin time (PT), ascites and hepatic encephalopathy) in the Child–Pugh score have no weight distinctions, and each indicator is greatly affected by other factors. The judgment of ascites and hepatic encephalopathy is subjective. The MELD score includes three indicators (TB, creatinine and international normalized ratio (INR)), which can overcome the influence of subjective factors. However, this score does not consider portal hypertension and complications, and some non-liver disease factors may also affect the TB, INR, and creatinine levels. Additionally, both scores are only used to evaluate whole liver function.

Gadolinium-ethoxybenzyl-diethylenetriamine penta-acetic acid (Gd-EOB-DTPA) is a hepatocellular contrast agent that is easily taken up by normal hepatocytes and secreted into the biliary system without any change in its chemical structure [1]. Additionally, Gd-EOB-DTPA has characteristics of both nonspecific extracellular space contrast agents and hepatocyte-specific contrast agents [2]. Therefore, Gd-EOB-DTPA-enhanced MR imaging has been used not only to detect and characterise liver lesions [3–6] but also for a one-stop assessment of hepatic function. Compared with the above two scoring systems, the greatest advantage of using MRI to evaluate liver function is that it can evaluate liver function at and below the liver segment. In addition to functional information, the data required for surgical planning, such as the tumour volume and distribution, liver anatomy, vascular supply, and related extrahepatic findings, can also be collected in one examination. Thus, it is possible to accurately predict the effective residual liver function after the operation and guide clinicians in adopting appropriate treatment plans for patients.

Previous studies have evaluated liver function through Gd-EOB-DTPA-enhanced MRI, including biliary tract enhancement [7], the liver signal intensity ratio with or without reference groups [8], T1 mapping [9], and dynamic contrast-enhanced MR imaging [10]. However, using the signal intensity ratio to assess liver function is the simplest and most convenient method. The relative enhancement ratio (RE) of the liver parenchyma and liver-to-spleen contrast ratio (LSC) has been widely described [11–13]. However, few studies have assessed liver function via the portal vein. Additionally, no study has indicated the best parameter that reflects liver function among the SI of the liver parenchyma, portal vein, and spleen and liver-to-portal vein (LPC), liver-to-spleen (LSC), portal vein-to-spleen (PSC) contrast ratios. Therefore, this study aimed to investigate whether these parameters can evaluate liver function in patients with cirrhosis and which is the best parameter.

## Methods

### Patients

This retrospective study of existing data was approved by the institutional review board, and the requirement for written informed consent was waived.

From November 2017 to October 2019, 761 Gd-EOB-DTPA-enhanced MR imaging examinations were performed. The exclusion criteria were as follows: liver function tests were not performed within 1 week before and after MR examination (n = 288); excessive motion artifacts or incomplete examination (n = 36); the main and branches of the portal vein were not visualized on MR images (n = 33); splenectomy or diffuse Gamna–Gandy bodies (n = 11); the presence of diffuse or massive (d > 10 cm) liver tumours, cysts and partial hepatectomy (n = 76); liver dysfunction without cirrhosis (n = 88); and various diseases of the biliary tract such as cholelithiasis or biliary duct dilatation (n = 109). Finally, the retrospective study comprised 41 normal subjects and 79 patients with liver cirrhosis [HBV-related cirrhosis (n = 52), HCV-related cirrhosis (n = 13), alcoholic cirrhosis (n = 5), and schistosomal cirrhosis (n = 9)].

### Clinical data

Two radiologists separately recorded the clinical data of the patients, including age, sex, biochemical tests associated with liver function (TB, direct bilirubin, indirect bilirubin, aspartate aminotransferase (AST), alanine aminotransferase (ALT), albumin, creatinine, platelet count, prothrombin time (PT), and INR) and clinical manifestations (ascites, hepatic encephalopathy). They also obtained the Child–Pugh and MELD scores. For all patients, Roche Cobas 8000 automatic chemistry analysis was used for biochemical tests. The two radiologists reviewed the records and reached an agreement.

### MR imaging

All examinations were performed using a 3-T MRI scanner (Ingenia, Philips Healthcare, Best, Netherlands) with a 32-channel body phased array coil as the receiving coil. Enhanced scanning was performed using a modified Dixon (mDixon) sequence. The parameters for mDixon were as follows: repetition time, 3.79 ms; echo time, 1.33 ms; sense factor, 2.0; flip angle, 18°; field of view, 352 × 400 mm; matrix, 268 × 236; reconstruction matrix, 400 × 400; bandwidth, 1260.6 Hz per pixel; scan time, 15 s; thickness, 5 mm. The HBP images were obtained 15 min after Gd-EOB-DTPA administration. Gd-EOB-DTPA (Primovist; Bayer Schering Pharma AG, Berlin, Germany) was used as a hepatocellular contrast agent. All the patients received the contrast agent injection at a rate of 1.0 mL/s (dose = 0.025 mmol/kg body weight). The contrast agent was intravenously administered via a power injector followed by a 25.0-mL saline flush.

### Imaging analysis

All examinations were reviewed by two other radiologists with 3 and 10 years of experience in abdominal MR imaging who were blinded to the patients’ clinical, laboratory, and radiological information. Regions of interest (ROIs) were drawn on the 15-min HBP images using a picture archiving and communication system (PACS; Neusoft, Shenyang, China). Each ROI was either a circle or an oval. The SI of the liver parenchyma was measured in four sections (left lateral, left medial, right anterior, and right posterior). The SI of the spleen parenchyma was measured in three evenly distributed sections. In each section of the liver and spleen, the ROI (ROI size: 200 mm^2^) was manually set by the observers, avoiding visible vessels and biliary ducts, focal lesions, and imaging artifacts. The portal vein ROIs were separately placed in the centre of the main portal and its right and left branches based on the location of the vessels in the portal phase. The size of each ROI depended on the diameter of the portal vein (ROI size: 8–40 mm^2^). LPC was calculated by dividing the liver parenchyma SI by the portal vein SI, LSC was calculated by dividing the liver parenchyma SI by the spleen SI, and PSC was calculated by dividing portal vein SI by the spleen SI as follows:

$${\text{LPC}} = [{\text{SI}}_{{{\text{liver}}}} ]/[{\text{SI}}_{{{\text{Portal vein}}}} ]$$

$${\text{LSC}} = [{\text{SI}}_{{{\text{liver}}}} ]/[{\text{SI}}_{{{\text{spleen}}}}]$$

$${\text{PSC}} = [{\text{SI}}_{{{\text{Portal vein}}}} ]/[{\text{SI}}_{{{\text{spleen}}}}]$$

### Statistical analysis

Statistical analysis was performed using IBM SPSS Statistics (version 25.0; Chicago, IL). The Kolmogorov–Smirnov test was used to evaluate the normality of the measurement data. Normally distributed data were presented as means ± standard deviation, and nonnormally distributed data were presented as medians (interquartile range). One-way ANOVA or the nonparametric Kruskal–Wallis test was used to compare the differences between the normal group and Child–Pugh class A, B, and C groups. One-way ANOVA with LSD or the rank-sum test with the Mann–Whitney U test was applied for analyses among groups. Spearman rank correlation coefficients were used to analyse the correlation between hepatic function laboratory markers and the MRI data of the 15-min HBP images. ROC analysis was used to discriminate between group 1 (normal group and Child–Pugh class A) and group 2 (Child–Pugh class B and C) on the 15-min HBP images. All the tests were two-sided, and* p* < 0. 05 indicated statistical significance.

## Results

### Clinical data and laboratory examination

The 120 included subjects comprised normal patients (n = 41) and patients with Child–Pugh class A (n = 50), B (n = 21) and C (n = 8) disease. The laboratory parameters and clinical data of the patients are shown in Table 1. No significant differences were found in age, sex, the creatinine level or the mean interval (between MRI and laboratory testing) among the groups. However, distinct differences were identified among all the analysed groups regarding TB, direct bilirubin, indirect bilirubin, albumin, ALT, AST, PLT, PT, INR, Child–Pugh score, and MELD score (*p* < 0.001).

**Table 1:** The laboratory parameters and clinical data of the patients

Characteristic	Total	Normal	C–P A	C–P B	C–P C	*P* value
Sample size	n = 120	n = 41	n = 50	n = 21	n = 8	–
Child–Pugh score	6.0 (5.0–7.0)		5.0 (5.0–6.0)	7.0 (7.0–8.0)	12.0 (11.3–12.0)	< 0.001
MELD score^a^	7.41 (6.43–8.52)	6.43 (6.43–6.43)	7.50 (7.12–7.97)	10.04 (8.55–10.50)	16.33 (15.34–16.69)	< 0.001
Mean interval(days)^a^	2.0 (0.3–4)	2.0 (1.0–4.0)	1.0 (0.0–3.3)	2.0 (0.5–3.0)	3.0 (1.3–5.0)	0.177
Age (years)^a^	56.0 (52.0–64.8)	54.0 (49.5–58.5)	60.0 (52.0–64.3)	60.0 (52.5–66.5)	55.0 (45.3–71.5)	0.208
Sex (male/female)	73/47	25/16	32/18	13/8	3/5	0.566
Standard hepatic function test						
TB (mg/dL)^a^	0.85 (0.60–1.18)	0.54 (0.40–0.65)	0.92 (0.80–1.09)	1.35 (1.13–1.88)	3.45 (3.13–3.95)	< 0.001
Direct bilirubin (mg/dL)^b^	0.49(0.13–2.38)	0.22 (0.13–0.30)	0.41 (0.20–0.75)	0.77 (0.56–1.37)	1.69 (1.28–2.38)	< 0.001
Indirect bilirubin (mg/dL)^b^	0.56 (0.15–1.97)	0.32 (0.15–0.53)	0.54(0.23–0.92)	0.87(0.23–1.97)	1.08 (0.72–162)	< 0.001
Albumin (g/dL)^a^	4.28 (3.9–4.57)	4.60 (4.41–4.71)	4.20 (4.10–4.40)	3.50 (2.97–3.82)	2.61 (2.39–2.64)	< 0.001
INR^a^	1.07 (0.99–1.15)	0.96 (0.95–1.00)	1.09 (1.05–1.12)	1.19 (1.15–1.24)	1.53 (1.47–1.67)	< 0.001
PT^a^	13.9 (12.8–14.6)	12.5 (12.3–12.8)	14.1 (13.8–14.5)	14.9 (14.4–15.8)	18.6 (18.1–19.2)	< 0.001
Serum hepatic enzyme levels						
AST (U/L)^a^	26.0 (18.0–38.0)	17.0 (14.0–21.0)	29.0 (21.0–37.3)	40.0 (34.5–61.0)	87.0 (70.3–92.8)	< 0.001
ALT (U/L)^a^	25.0 (17.0–35.8)	17.0 (11.5–21.0)	27.0 (21.0–37.3)	39.0 (34.0–42.5)	71.0 (63.3–84.3)	< 0.001
Serum renal function levels						
Creatinine (mg/dL)^a^	0.73 (0.67–0.82)	0.71 (0.65–0.77)	0.76 (0.69–0.90)	0.72 (0.64–0.78)	0.76 (0.70–0.95)	0.132
Platelet count^a^	135 (81.0–209.8)	230.0 (182.0–262.0)	124.5 (84.8–143.0)	78.0 (68.5–90.0)	48.5 (36.8–66.8)	< 0.001

MELD, model for end-stage liver disease; TB, total bilirubin; INR, international normalized ratio; PT, Prothrombin time; AST, aspartate transaminase; ALT, alanine transaminase

^a^Data is presented as median (interquartile range)

^b^Data is presented as mean (minimum and maximum)

### Differences in the MRI data among the groups on 15-min HBP images

**Fig. 1:**
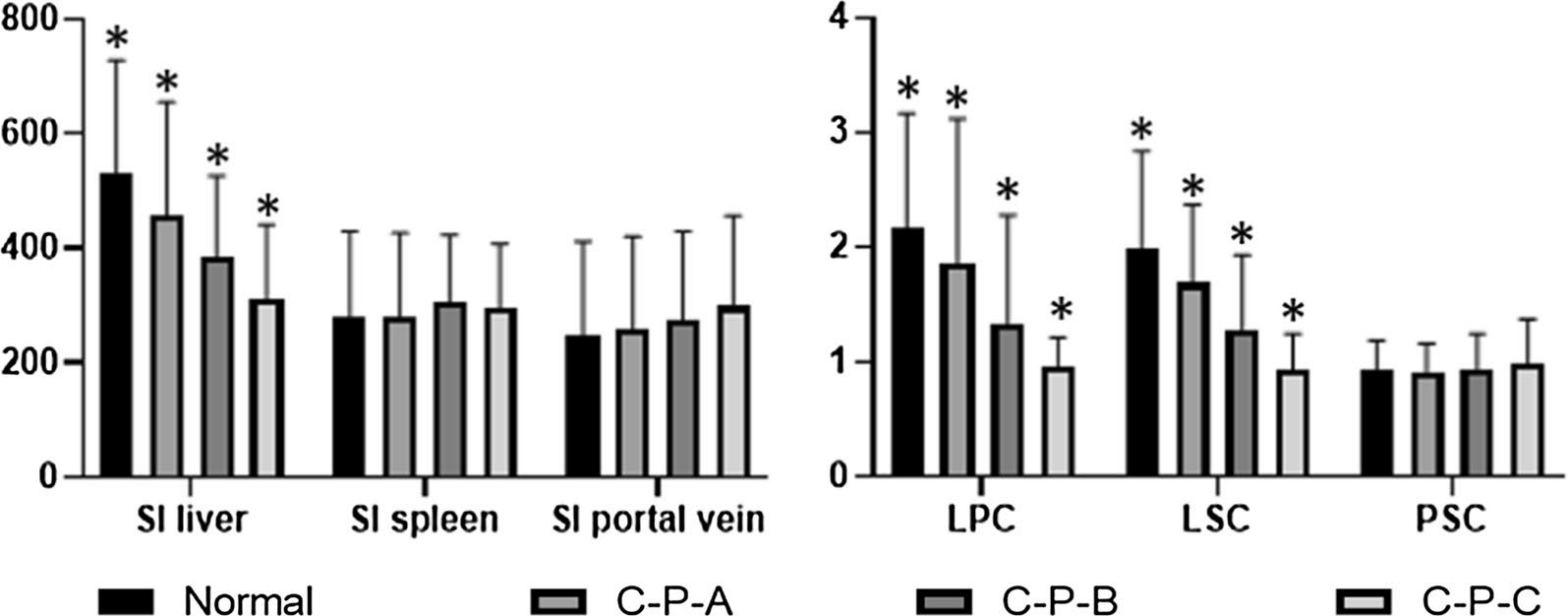
Differences in the MRI data among the groups on 15-min HBP images. The data were presented as means ± 2SD. *Statistical differences among the groups (*P* < 0.05)

The liver parenchyma SI, LPC and LSC values were significantly different among the groups on the 15-min HBP images (*p* < 0.001), and these values gradually decreased from normal to Child–Pugh C cirrhotic livers. The spleen parenchyma SI, portal vein SI and PSC values were not significantly different among the groups, and the portal vein SI constantly and slightly increased from normal to Child–Pugh class C (*p* > 0.05) cirrhotic livers (Fig. 1).

### Correlation between the laboratory parameters and MRI data

**Fig. 2:**
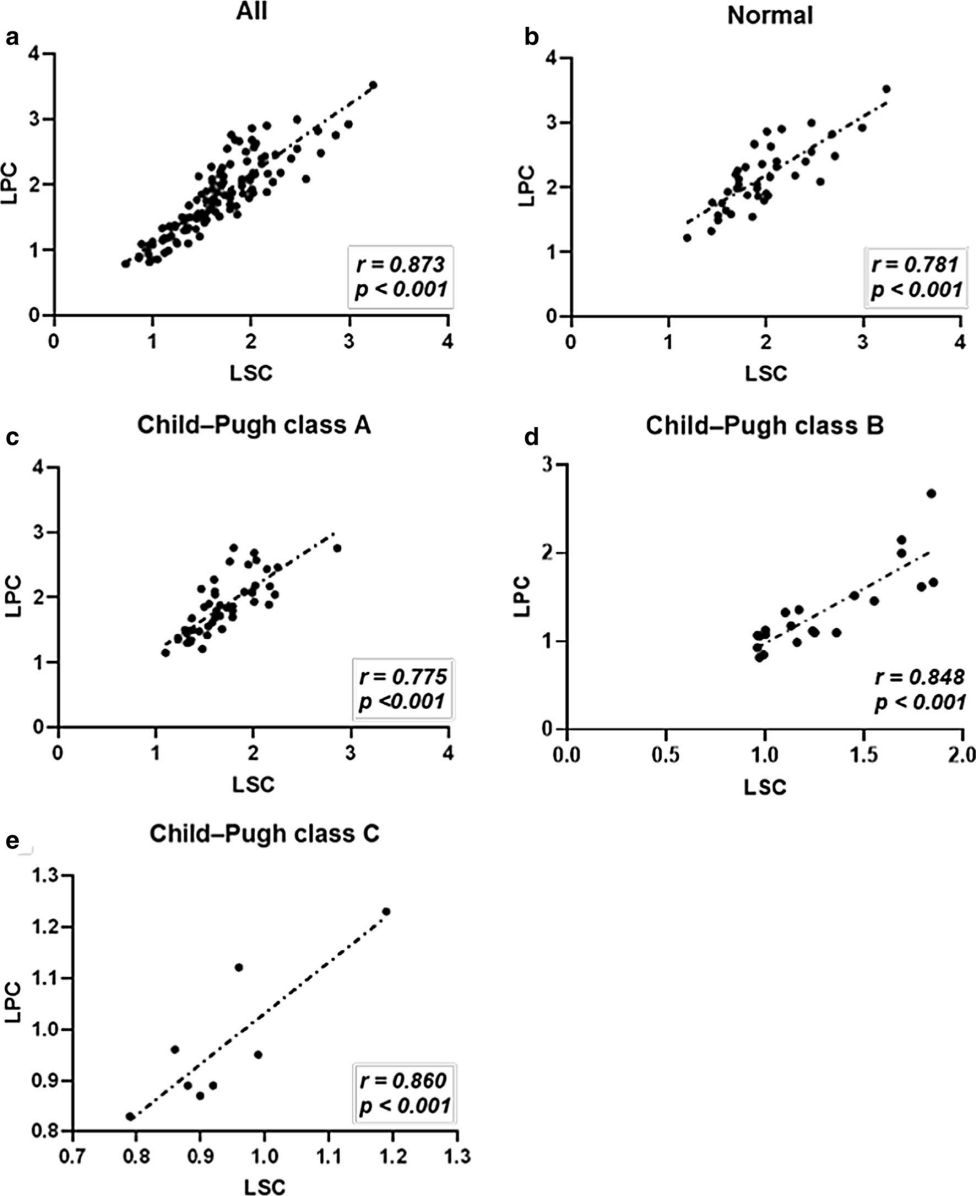
Correlations between LSC and LPC among the groups: **a** overall, **b** normal, **c** Child–Pugh A, **d** Child–Pugh B and **e** Child–Pugh C. These correlations were strongly positive

The correlations between the laboratory parameters and MRI data on the 15-min HBP images are summarized in Table 2. TB, albumin, PT, INR, PLT, ALT, AST, Child–Pugh score, and MELD score were significantly correlated with liver parenchyma SI, LPC and LSC. A strong correlation was observed between LPC and LSC in all the groups (Fig. 2).

**Table 2:** The correlation among the liver function parameters, liver function scores and MRI data

Laboratory indexes	Correlation coefficient			*P* value
	LPC	LSC	SI_liver_	
TB	− 0.577	− 0.613	− 0.522	< 0.001
Albumin	0.565	0.623	0.479	< 0.001
ALT	− 0.426	− 0.455	− 0.492	< 0.001
AST	− 0.477	− 0.512	− 0.506	< 0.001
INR	− 0.641	− 0.646	− 0.553	< 0.001
PT	− 0.579	− 0.576	− 0.524	< 0.001
Creatinine	− 0.090	− 0.139	0.024	> 0.050
Platelet count	0.464	0.467	0.518	< 0.001
Child–Pugh score	− 0.576	− 0.569	− 0.562	< 0.001
MELD score	− 0.632	− 0.580	− 0.526	< 0.001

TB, total bilirubin; ALT, alanine transaminase; AST, aspartate transaminase; INR, international normalized ratio; PT, Prothrombin time; MELD, model for end-stage liver disease

### ROC analysis

**Fig. 3:**
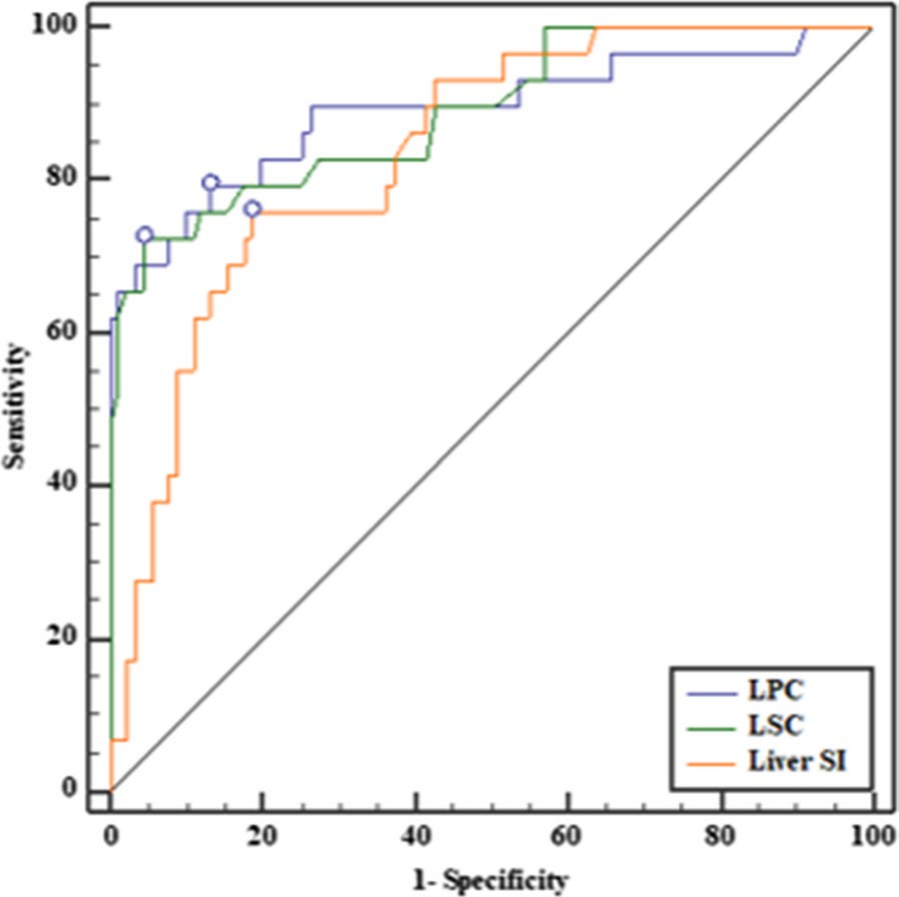
ROC curve analysis showed that the AUC value of LPC was 0.892 (95% CI 0.822–0.941), that of LSC was 0.889 (95% CI 0.818–0.939), and that of the liver parenchyma SI was 0.836 (95% CI 0.758–0.898)

ROC analysis revealed that the optimal cut-off value for LPC to distinguish group 1 (normal group and Child–Pugh class A) from group 2 (Child–Pugh class B and C) was 1.20 (AUC 0.892), with a sensitivity of 98.9% and a specificity of 69.0%. The optimal cut-off value for LSC to distinguish group 1 from group 2 was 1.27 (AUC: 0.889), with a sensitivity of 95.6% and a specificity of 72.4%. The optimal cut-off value for the liver parenchyma SI to distinguish group 1 from group 2 was 405.4 (AUC: 0.836), with a sensitivity of 81.3% and a specificity of 75.9% (Fig. 3). The differences in AUCs among LPC, LSC and liver parenchyma SI were not significant (*p* > *0.05*). The reason for this grouping was that patients with Child–Pugh class B or C cirrhosis had contraindications to surgery [14, 15].

## Discussion

Cirrhosis can damage liver cells, increase the spleen volume, and lead to portal hypertension. Therefore, we mainly assessed the liver, spleen and portal vein. Our research was conducted using 15-min HBP images, and we believed that this period could meet the needs to diagnose liver diseases and shorten the examination time of patients.

The liver parenchyma SI can be used to estimate liver function, which has been widely described. The hepatobiliary phase of Gd-EOB-DTPA-enhanced images is due to the selective uptake of membrane-bound organic anion transporters (OATP1 B1/B3) [16–18]. Normal hepatocytes can use these transporters to take up Gd-EOB-DTPA, and the amount of Gd-EOB-DTPA peaked on the 20-min HBP images; the number of impaired transporters and functional capacity of these transporters could reduce the uptake of Gd-EOB-DTPA into hepatocytes [19], subsequently affecting the liver signal. Our data showed that the liver parenchyma SI gradually decreased with increasing liver function damage. Previous studies [19–21] have also indicated that the severity of cirrhosis can significantly affect the absorption of gadolinium and then affect the degree of liver enhancement, a finding that was consistent with ours.

**Fig. 4:**
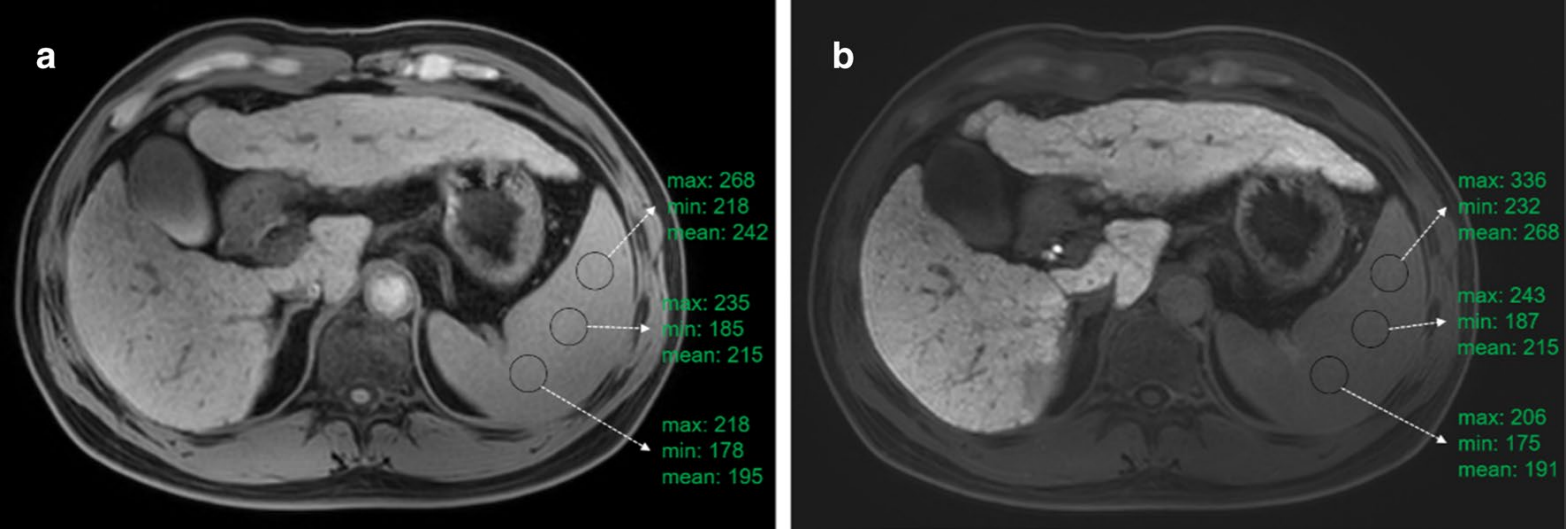
A case of liver cirrhosis with an uneven spleen signal: **a** pro-enhanced image and **b** hepatobiliary phase at 15 min image. ROI size: 200 mm × mm

The spleen does not contain the organic anion transporters described above, and Gd-EOB-DTPA only shows the characteristics of a nonspecific extracellular space contrast agent. Our data indicate that the SI of spleen cannot reflect liver function, and the mean value of the spleen signal is equally likely in each group. Additionally, we found that, in most cases in this study, the spleen signal increased gradually from right to left on both pro-enhanced images and 15-min HBP images (Fig. 4), leading to an increase in the mean signal value of the spleen. The cause remains unclear and may be related to the uneven magnetic field or hemodynamic of the spleen.

**Fig. 5:**
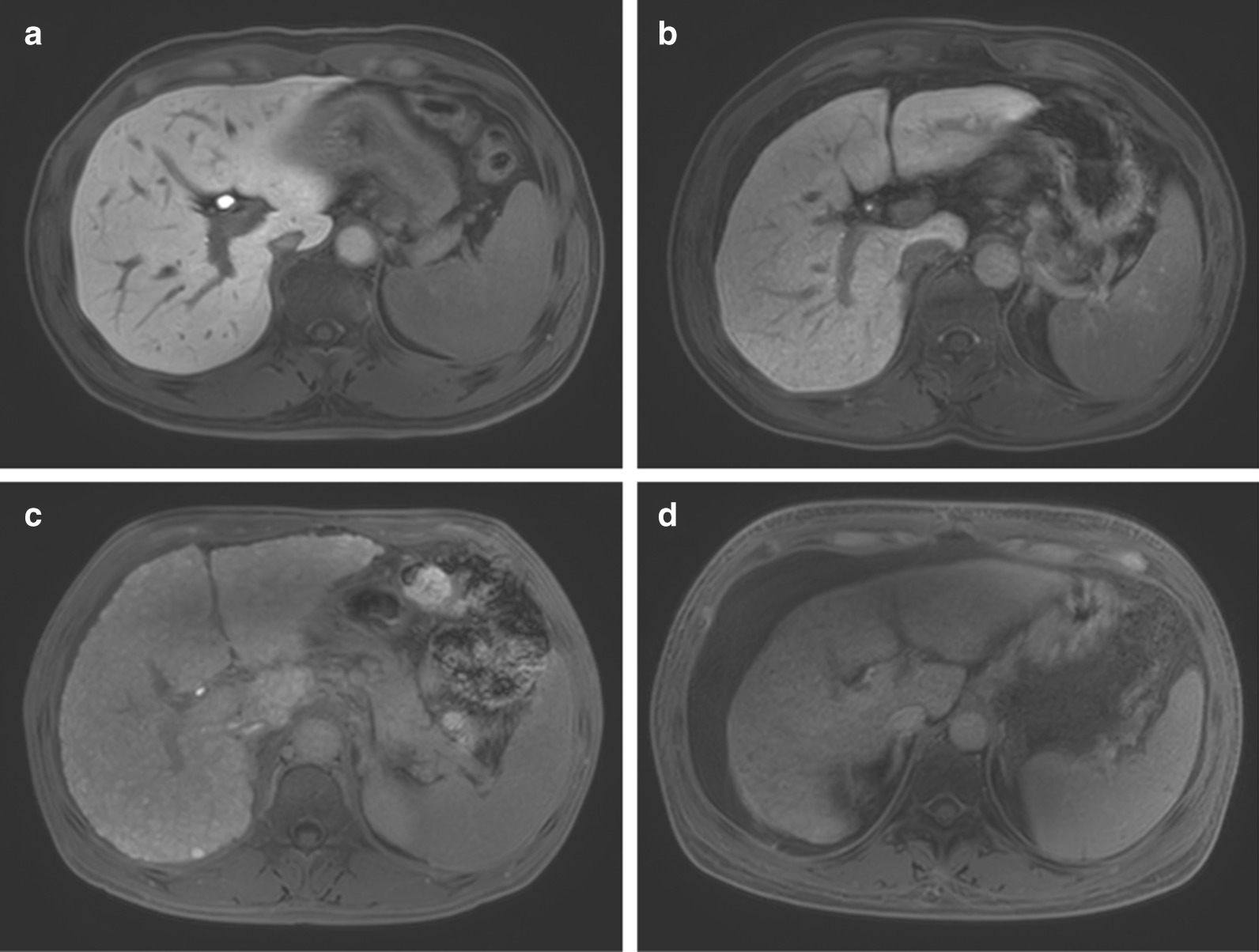
Hepatobiliary phase in 15-min images among the groups: **a** normal, **b** Child–Pugh A, **c** Child–Pugh B and **d** Child–Pugh C. The direct bilirubin of this patient was 2.38 mg/dl

In our study, the portal vein SI constantly and slightly increased from normal livers to Child–Pugh class C cirrhotic livers, but no difference was found among the groups. Zhang reported that LPC could effectively indicate the severity of liver function [22], and their data on portal vein SI are similar to ours. A previous study suggested that the delayed hyperintensity in the portal vein can potentially be used to reflect hepatobiliary function [23]; however, the subjects in that study were mostly patients with extrahepatic cholestasis. We found no delayed hyperintensity in the portal vein in any of the subjects in our study, and the direct bilirubin levels in all the groups were lower than the cut-off value of 2.18 mg/dl, except for one patient in group C (2.38 mg/dl). We think that is the main reason for the difference between studies. The hepatobiliary phase images among the groups are shown in Fig. 5. A study proved that hepatic uptake and biliary elimination of bilirubin compete against Gd-EOB-DTPA uptake, and hyperbilirubinemia will lead to the decreased absorption and clearance of Gd-EOB-DTPA, which can also cause delayed contrast agent clearance from the blood [24]. However, we hold that the bilirubin level in patients with cirrhosis may not be as high as that in patients with extrahepatic cholestasis, and hepatocytes may withstand this competition in patients with cirrhosis.

Unlike that of enhanced CT, the signal intensity of enhanced MRI has a nonlinear relationship with the contrast agent concentration, and most studies have used a reference tissue (spleen) to correct the liver signal. Only one study has examined the relationship between LPC and LSC [25]. Their results showed that LPC strongly correlates with LSC, and the LPC of each group was lower than that of LSC. The authors believed that the cause might be the portal vein SI, which can more reflect the blood pool than the spleen. Our research also showed a strong correlation between LPC and LSC among the groups, but LPC was greater than LSC. The reasons for this difference may be as follows: (1) different causes might have led to the different patterns of uptake and excretion of Gd-EOB-DTPA: our patients mainly had hepatitis B cirrhosis, and their patients mainly had chronic liver disease; and (2) the MRI devices and imaging sequences were different.

To our best knowledge, no study has investigated the value of PSC in evaluating liver function in cirrhosis. Our research proved, for the first time, that PSC cannot reflect liver function in patients with cirrhosis. As discussed previously, the portal vein SI constantly and slightly increased from normal livers to Child–Pugh class C cirrhotic livers, but no differences were found among the groups, and the mean value of the spleen signals was likely equal across the groups. No difference in PSC may exist among the groups.

Some studies have used ICG to reflect liver function because a direct correlation has been found between ICG clearance and hepatocytes, and this parameter can provide more complete information on liver uptake and excretion function [26–28]. We did not analyse ICG because of operational difficulties. We quantitatively analysed the correlations between the MRI data and liver function parameters. In this study, the liver parenchyma SI, LPC and LSC were weakly to moderately correlated with laboratory markers. Zhang also demonstrated a weak to moderate correlation between LPC and laboratory markers [22], consistent with our findings. We also found that the liver parenchyma SI, LPC and LSC were negatively correlated with hepatic function scores (Child–Pugh score and MELD score), and the correlation coefficients of the parameters, in order from the largest to smallest, was as follows: LPC, LSC, and the liver parenchyma SI. The cause may be that the changing trend of the portal vein signal strengthens the correlation between LPC and liver function.

Receiver operating characteristic analysis showed that the order of the AUCs of the parameters, from the largest to smallest, was as follows: LPC, LSC, and the liver parenchyma SI (0.892, 0.889, and 0.836, respectively). However, the differences in AUCs among LPC, LSC and the liver parenchyma SI were not significant. Thus, these parameters have the same ability to distinguish between group 1 and group 2.

These results suggest that LPC may be a more useful alternative imaging biomarker to evaluate liver function than LSC and the liver parenchyma SI. Takatsu found that LPC could be used as a substitute for LSC for a simple assessment of the degree of hepatic contrast enhancement [25], consistent with our findings. Additionally, the authors believed that LPC could be particularly useful in cases of splenectomy and Gamna–Gandy bodies [25]. However, we thought this conclusion needed further verification because of the small number of patients who had undergone splenectomy (n = 6) and those with Gamna–Gandy bodies (n = 7), and all of these patients had Child–Pugh class B disease.

Additionally, nuclear medicine tracers that assess liver function have been reported, mainly ^99m^Tc-galactosyl human serum albumin (GSA) and ^99m^Tc-mebrofenin. GSA is an asialoglycoprotein analogue, and mebrofenin is an iminodiacetic acid (IDA) analogue [29]. The tracers ^99m^Tc-GSA and ^99m^Tc-mebrofenin can be specifically absorbed by hepatocytes after injection into the body. The combination of SPECT and CT allows for 3D distribution analysis and more precise measurements. Therefore, these tracers can be used to accurately and quantitatively analyse the liver function reserve of each liver segment. However, the disadvantages are obvious, such as the fusion method of SPECT images and CT images not being standardized, radiation exposure and low image resolution. Rassam et al. compared dynamic gadoxetate-enhanced MRI and ^99m^Tc-mebrofenin hepatobiliary scintigraphy with SPECT to assess liver function and found that the mebrofenin uptake rate (MUR) and mean Gd-EOB-DTPA uptake rate (KI) of the whole liver correlated strongly with liver function and that a moderate correlation exists between RE and MUR [30]. Geisel et al. also found that RE and the hepatic uptake index (HUI) correlate with MUR [31]. These studies suggest that the assessment of liver function with Gd-EOB-DTPA MRI is comparable to imaging with ^99m^Tc-mebrofenin or GSA. Compared with the signal intensity, quantitative parameters such as KI, T1 values and T2* values (obtained from T1 mapping [9] and T2*mapping [32], respectively) can reflect liver function more accurately, but the data acquisition obstacles, uncertainty of the optimum pharmacokinetic model and most suitable parameters might limit their application. Nevertheless, these results indicate that GD-EOB-DTPA MRI is an ideal choice for preoperative liver function evaluation.

Our study had several limitations. First, the severity of cirrhosis was not grouped based on liver biopsy results. Second, we did not classify the causes of cirrhosis, and different causes might lead to different patterns of uptake and excretion of Gd-EOB-DTPA. Third, it was difficult to avoid selection bias because of the retrospective nature of this study. Fourth, this study included a small number of patients with Child–Pugh class C disease who have a poor physical condition and decompensated cirrhosis and cannot undergo the examination. Thus, further prospective and multicentre studies that include more patients with Child–Pugh class C disease are needed and that classify the causes of cirrhosis. Finally, this study only evaluated whole liver function. Clinically, segmental liver function is more meaningful than whole liver function. Therefore, we will measure and explore segmental liver function according to liver segment in the future.

## Conclusion

Liver function can be assessed and classified using LPC, LSC and the liver parenchyma SI obtained in the hepatobiliary phase of Gd-EOB-DTPA-enhanced MRI. LPC might be a more useful imaging biomarker to evaluate liver function than LSC and the liver parenchyma SI. Additionally, the portal vein SI showed a certain increasing trend with the aggravation of liver function damage, but it cannot reflect the liver function of patients with cirrhosis. PSC also cannot reflect liver function.

## Data Availability

Data related to the current study are available from the corresponding author on reasonable request.

## Abbreviations

Gd-EOB-DTPA: Gadolinium-ethoxybenzyl-diethylenetriamine penta-acetic acid
MRI: Magnetic resonance imaging
LPC: Liver-to-portal vein contrast ratio
LSC: Liver-to-spleen contrast ratio
PSC: Portal vein-to-spleen contrast ratio
SI: Signal intensity
AUC: Area under curve
ROI: Region-of-interest
HBP: Hepatobiliary phase
MELD: Model for end-stage liver disease
TB: Total bilirubin
INR: International normalized ratio
PT: Prothrombin time
AST: Aspartate transaminase
ALT: Alanine transaminase
GSA: Galactosyl human serum albumin
IDA: Iminodiacetic acid
SPECT: Single photon emission computed tomography
CT: Computed tomnography
MUR: Mebrofenin uptake rate
KI: The mean Gd-EOB-DTPA uptake rate
HUI: Hepatic uptake index
